# Impacts of Environmental Heterogeneity on Moss Diversity and Distribution of *Didymodon* (Pottiaceae) in Tibet, China

**DOI:** 10.1371/journal.pone.0132346

**Published:** 2015-07-16

**Authors:** Shanshan Song, Xuehua Liu, Xueliang Bai, Yanbin Jiang, Xianzhou Zhang, Chengqun Yu, Xiaoming Shao

**Affiliations:** 1 College of Biological Sciences, China Agricultural University, Beijing, China; 2 School of Environment, Tsinghua University, Beijing, China; 3 College of Life Science, Inner Mongolia University, Hohhot, China; 4 College of Resources and Environment, Huazhong Agricultural University, Wuhan, China; 5 Institute of Geographic Sciences and Natural Resources Research, Chinese Academy of Sciences, Beijing, China; 6 Beijing Key Laboratory of Biodiversity and Organic Farming, College of Resources and Environmental Sciences, China Agricultural University, Beijing, China; Tennessee State University, UNITED STATES

## Abstract

Tibet makes up the majority of the Qinghai-Tibet Plateau, often referred to as the roof of the world. Its complex landforms, physiognomy, and climate create a special heterogeneous environment for mosses. Each moss species inhabits its own habitat and ecological niche. This, in combination with its sensitivity to environmental change, makes moss species distribution a useful indicator of vegetation alteration and climate change. This study aimed to characterize the diversity and distribution of *Didymodon* (Pottiaceae) in Tibet, and model the potential distribution of its species. A total of 221 sample plots, each with a size of 10 × 10 m and located at different altitudes, were investigated across all vegetation types. Of these, the 181 plots in which *Didymodon* species were found were used to conduct analyses and modeling. Three noteworthy results were obtained. First, a total of 22 species of *Didymodon* were identified. Among these, *Didymodon rigidulus *var. *subulatus* had not previously been recorded in China, and *Didymodon constrictus *var. *constrictus* was the dominant species. Second, analysis of the relationships between species distributions and environmental factors using canonical correspondence analysis revealed that vegetation cover and altitude were the main factors affecting the distribution of *Didymodon* in Tibet. Third, based on the environmental factors of bioclimate, topography and vegetation, the distribution of *Didymodon* was predicted throughout Tibet at a spatial resolution of 1 km, using the presence-only MaxEnt model. Climatic variables were the key factors in the model. We conclude that the environment plays a significant role in moss diversity and distribution. Based on our research findings, we recommend that future studies should focus on the impacts of climate change on the distribution and conservation of *Didymodon*.

## Introduction

As an important component of ecosystems, mosses have a strong influence on the cycling of water, nutrients, energy, and carbon [1,2]. Given their sensitivity to environmental change, mosses can be used as bioindicators of forest integrity [3], water quality [4], air pollution [5], metal accumulation [6], and climate change [7,8]. They also play key roles in long-term processes such as peat accumulation, the formation of microtopography, and permafrost stability [1]. Although mosses are widely distributed on land, different types of moss have certain ranges of climate and environmental conditions within which they can survive and successfully reproduce. Topographic factors such as aspect, slope, and altitude [9,10]; climatic factors [11]; soil factors including type, moisture, and pH [12,13]; vegetation type and coverage; and the type of substrate that mosses grow on [11,12,14] are all important environmental factors affecting the distribution of mosses.

*Didymodon* is the largest genus in the Pottiaceae family and includes approximately 122 species that are distributed worldwide, with the greatest diversity found in temperate and mountainous regions, where they primarily grow on rocks or soil [15]. The species diversity varies greatly in regions with a wide variety of habitats and high level of environmental heterogeneity. Although studies of *Didymodon* date back to the year 1801, only recently has the exact phylogenetic position of this genus been established. Species of *Didymodon* are often distributed on calcareous soil or rocky areas [16]. In China, a total of 26 species of *Didymodon* have been reported [17]. Many of these species are widely distributed, whereas some are found only in specific areas; for example, *Didymodon anserinocapitatus* is found only in Tibet, and *Didymodon rigidulus* var. *icmadophilus* occurs only in Inner Mongolia [18]. Currently, researchers studying *Didymodon* are paying close attention to its taxonomic [19,20], systematic [21], and morphological characters [22,23], but few are conducting research on the distribution of *Didymodon* with a consideration of habits and environmental variables at large scales.

The Tibetan Plateau has been called the roof of the world because of its very high altitude [24,25]. Characterized by its extreme environments, Tibet is particularly at risk from vegetation changes due to climatic change. Given the relatively low intensity of human disturbance occurring in the region, and the unique vegetation and climate zones of the plateau, there have been many studies of the effects of climate change on ecosystems in Tibet [26]. The first moss specimen was collected by Thomson during 1847–1849 in western Tibet. In China, comprehensive contributions were primarily made by scientific expeditions to the Qinghai-Tibet Plateau in 1952–1979 [27]. In recent years, further studies on moss ecology have been carried out, considering subjects such as the accumulation of heavy metals, responses to changes in air humidity, and bryophyte communities in the valley areas of Tibet [28,29,30]. Because of geographical conditions and traffic restrictions, these surveys have been concentrated in southeastern Tibet, and the northwest regions have rarely been investigated. However, although previous studies have focused on moss taxonomy, the environmental factors that affect moss diversity and distribution at microhabitat and macro spatial scales are still unknown [27].

Given its resistance to cold and drought, *Didymodon* is the dominant genus of moss in Tibet. *Didymodon* exhibits apparent morphological, physiological, and genealogical adaptations to its particular environments [15]. The Tibetan Plateau is an important area for the study of climate change and its effect on vegetation at large scales. Thus, understanding the diversity of *Didymodon* species, as well as their spatial distribution patterns and related environmental factors, is helpful for protecting Tibetan ecosystems, monitoring changes in vegetation and climate, and guiding future field surveys of unexplored areas in Tibet.

In recent years, remote sensing, geographical information systems (GIS), and species distribution models have been introduced for the study of moss distributions at large spatial scales. Rapalee et al. [31] used advanced, very high resolution radiometer data to simulate the distribution of mosses in the boreal forest ecosystem of central Canada, then compared their different distributions at scales of 10 m, 30 m, and 1 km. Vanderpoorten et al. [32] studied the growth of the rare moss *Aneura maxima* using GIS, and then predicted its distribution. Jiang et al. [33] predicted the distribution of epiphyllous liverworts in China based on environmental variables, using the MaxEnt model. However, these studies did not focus on the spatial distribution of *Didymodon* or its relationship to environmental factors in Tibet.

The objectives of this study were as follows: (1) to survey the species richness and diversity of *Didymodon* in Tibet, (2) to analyze the relationship between species distribution and micro-habitat environments, and (3) to identify macro-habitat factors affecting the spatial distribution of *Didymodon* on a broad spatial scale.

## Materials and Methods

### Study area

**Fig 1:**
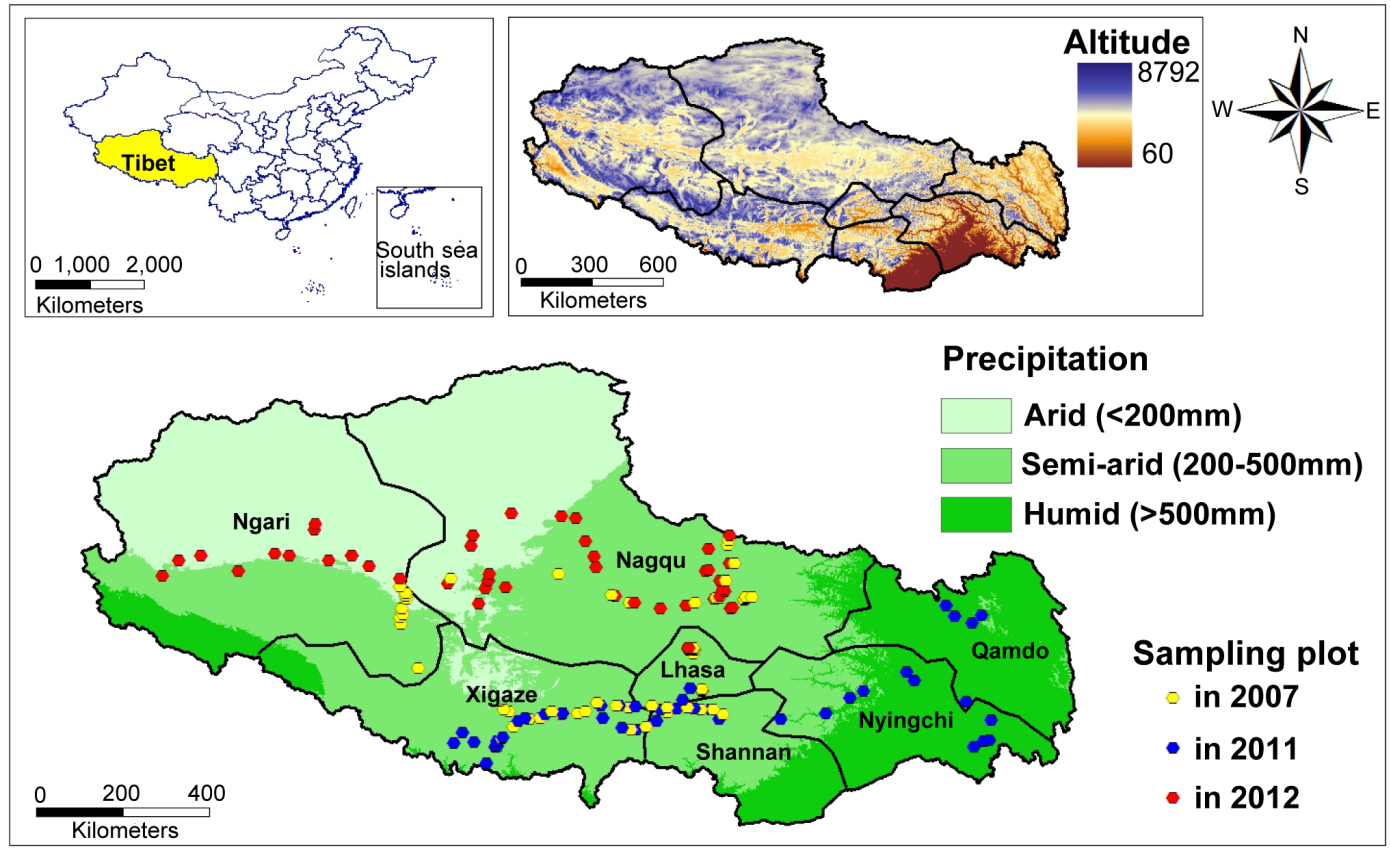
The study area containing 221 sampling plots, which was investigated in 2007, 2011, and 2012. Pale green represents arid areas that receive an annual precipitation of less than 200 mm; medium green indicates semiarid regions, where the annual precipitation is between 200 and 500 mm; and dark green indicates humid areas, where the annual precipitation is greater than 500 mm.

Tibet is located on the highest and largest plateau on Earth. Located in southwestern China (E78°25′–99°06′, N26°50′–36°53′), it is known as the world's third pole and the roof of the world (Fig 1). Tibet covers 1.2 million km^2^, and makes up 12.5% of the total area of China. The Tibetan region, which has an average altitude in excess of 4000 m, slopes gently downward from the northwest to the southeast. The region is surrounded by the Himalayas and the Kunlun and Tanggula Mountains, with an average altitude of over 6000 m. Tibet is also the source region of many major rivers, such as the Mekong, Indus, and Brahmaputra [24].

Its complex landforms and physiognomy form a unique array of high altitude climates, characterized by strong solar radiation, low temperatures with high daily temperature variance, distinct rainy and dry seasons, low air pressure, low ambient oxygen levels, and high winds. The vegetation in this area is characterized by zonal perpendicularity and diversification. Annual rainfall varies greatly within the study area; most areas receive less than 500 mm, including northern Tibet, the entirety of Ngari and Nagqu, the midwestern region of Xigaze, and part of Shannan, Lhasa, and Qamdo. The elevation in this region ranges from 60 to 8792 m. The major vegetation types are saline land, mountain meadow, mountain steppe, desert steppe, swamp meadow, and alpine-cold meadow.

The national fundamental geographic data, which include spatial data concerning boundaries, rivers, roads, and other features, were obtained from the National Geomatics Center of China (http://www.cehui8.com/3S/GIS/20130702/205.html). We used ArcGIS 10.0 (Esri, Redlands, CA, USA) to select and export the shapefile of Tibet from China. The shapefiles were deposited in Dryad (http://datadryad.org/review?doi=doi:10.5061/dryad.m8b96).

### Field sampling

Fieldwork was carried out from July to September in 2007, 2011, and 2012. The 221 sample plots were located at various altitudes in the arid, semi-arid, and humid regions of Tibet, in order to ensure that all vegetation types (11 groups of vegetation types, including alpine meadow, mountain steppe, pine forest, shrubbery, alpine tundra, deciduous broadleaved forest, desert steppe, swamp meadow, saline land, artificial woodland and alpine vegetation) were represented (Fig 1). The sample plots were situated at 100 m altitudinal intervals in the Himalayas, Tanggula Mountains, and Nyainqêntanglha Mountains, thus covering a significant elevation gradient. Finally, 181 plots in which *Didymodon* was found were used to conduct diversity analyses and distribution modeling. In each sample plot, three to seven quadrats (1 m^2^ area) were used; in total, 995 quadrats were used to investigate all ground-moss species [34]. Moss coverage was measured using a metallic quadrat divided into 100 grid cells and the number of grid cells that mosses covered was recorded. Moss specimens were collected from the sites, air dried, and identified to species level in the laboratory. Department of Science &Technology of Tibet Autonomous Region approved the field study, and none of the protected species was sampled during this study.

### Species identification and diversity analysis

Using the characteristics of the gametophytes (plants, stems, leaves, specialized asexual reproduction, perichaetia, color reactions of leaf cells in 2% potassium hydroxide solution) and sporophytes (seta, capsule, calyptras, spores), each specimen was identified to genus and species in the laboratory, using a stereomicroscope and an optical microscope.

Species diversity was analyzed using relative frequency, relative coverage, and importance value. The following indices were used in this study [35].

Frequency: F = f/T×100 (1)

$$Relative\;frequency:\;RF\;=\;F_{i}/\sum_{i\;=\;1}^{F}F\times 100$$ (2)

where *f* is the number of sample plots in which a moss species appears, *T* is the total number of sample plots, and *F*_*i*_ is the relative frequency of species *i*.

$$Relative\;coverage:\;RC\;=\;C_{i}/\sum_{i\;=\;1}^{C}C\times 100$$ (3)

where *C*_*i*_ is the coverage of species *i*, and *C* is the total coverage of species in each sampling plot.

Important value index: IV = (RC+RF)/2 (4)

The analyses were performed using the IBM SPSS Statistics (v19.0) software program, which was downloaded from the website http://emuch.net/html/f188.html.

### Environmental variables

Detailed information about habitat characteristics were recorded during the collection of species data in the sampling plots. Altitude was measured by a GPSMap60 CSx (Garmin Corporation, Shijr, Taiwan). Vegetation cover was estimated visually as a projection of plant cover. The substrate that mosses grow on was divided into six types: land, stone, tree, water, rock cracks, and thin soil layer above the rock. Temperature and humidity were measured with a PH-II-C handheld weather station. Soil moisture was measured with a FieldScout TDR100 Soil Moisture Meter (Spectrum Technologies, Inc., Plainfield, IL, USA) at soil depths of 3.8 and 7.6 cm. The variables used in analyzing the effects of environmental heterogeneity are shown in Table 1.

**Table 1:** Environmental variables used for correlation analyses between environmental variables and *Didymodon* diversity in the study area.

Category	Variables	Abbreviation	Units
Topographic	Altitude	Altitude	M
Vegetation	Vegetation type	Veg-type	dimensionless
	Vegetation cover	Veg-cove	Degree
Substrate	Substrate	Substrate	Degree
Bioclimatic	Temperature	Temp	°C
	Humidity	Humidity	dimensionless
Soil	Soil moisture 3.8	Soil-mois	dimensionless
	Soil moisture 7.6	Soil-mois2	dimensionless

In this study, three categories of spatial data with a total of 18 GIS layers of large-scale environmental variables (Table 2) were collected in order to facilitate modeling of the distribution of *Didymodon* in Tibet, using the MaxEnt model.

**Table 2:** Environmental variables used in modeling the distribution of *Didymodon* in the study area.

Category	Variables	Abbreviation	Units
Bioclimatic	Annual Mean Temperature	t_mean	°C
	Temperature Seasonality	t_seas	dimensionless
	Max Temperature of Warmest Month	t_max	°C
	Min Temperature of Coldest Month	t_min	°C
	Annual Precipitation	p_ap	mm
	Precipitation in Wettest Month	p_max	mm
	Precipitation in Driest Month	p_min	mm
	Precipitation Seasonality	p_seas	dimensionless
	Potential evapotranspiration	PET	mm
	Aridity	AI	dimensionless
Topographic	Altitude	Altitude	m
	Aspect	Aspect	degree
	Slope	Slope	degree
Vegetation	Annual minimum NDVI	NDVI_min	dimensionless
	Annual mean NDVI	NDVI_mean	dimensionless
	Annual maximum NDVI	NDVI_max	dimensionless
	Amplitude NDVI	NDVI_amp	dimensionless
	Standard deviation NDVI	NDVI_std	Dimensionless

1. Temperature and precipitation data were acquired from the database of the Chinese Meteorological Administration (http://www.cma.gov.cn/2011qxfw/2011qsjcx/). We selected records from 200 stations with less than 5.00% missing data between 2003 and 2012. Data concerning annual time series with annual means, seasonality, and extreme or limiting temperature and precipitation data were used [33,36]. The selected climate records were then interpolated to spatial climate datasets with a grain size of 1 × 1 km, using the thin-plate smoothing spline interpolation method of the ANUsplin software package [37]. Potential evapotranspiration (PET) and aridity index (AI) values were obtained from the CGIAR-CSI GeoPortal (http://csi.cgiar.org);
2. Digital elevation model data were obtained from the USGS GTOPO30 series (http://www1.gsi.go.jp/geowww/globalmap-gsi/gtopo30/gtopo30.html) and used to derive the aspect and slope data using ArcGIS 10.0;
3. Normalized difference vegetation index (NDVI) images at a 1-km resolution were obtained from Spot-Vegetation Programme (www.vgt.vito.be). The images were 10-day composites obtained by vegetation sensors located on SPOT4 and SPOT5 satellites. Using ERDAS IMAGINE software (Hexagon Geospatial, Norcross, GA, USA), we obtained NDVI indices from 2010 to 2012.

All of the environmental variables were projected as GIS raster layers in GCS_WGS_1984 coordinate system, and converted to ASCII format for using in the MaxEnt model, with a spatial resolution of 1 km. The data used in MaxEnt model was deposited in Dryad (http://datadryad.org/review?doi=doi:10.5061/dryad.m8b96).

### Habitat heterogeneity analysis

At the microhabitat scale, habitat heterogeneity analysis was used to explore the relationships (1) between habitat properties and species richness, and (2) between habitat properties and species composition. Using canonical correspondence analysis (CCA) to analyze the relationship between *Didymodon* diversity and habitat properties required two data matrices (S1 File): one was the *Didymodon* matrix, which contained species names and coverages (**Table A in** S1 File); the other was an environmental data matrix, including all the information for quadrats in which *Didymodon* mosses were present (**Table B in** S1 File). Multiple environmental factors were analyzed together in the CCA, which was performed using CANOCO for Windows 4.5 (downloaded from http://download.csdn.net/detail/slowslap/1556879), and the relationships between species diversity and micro-environmental variables were displayed using CANODRAW.

In addition to CCA, CANOCO for Windows 4.5 software was also used to perform correlation analyses of different habitat properties, which necessitated the analysis of variables that were not normally distributed. We reported significant correlations at p < 0.05 or p < 0.01.

### MaxEnt modeling

The MaxEnt algorithm in ecological niche modeling is a general-purpose machine-learning method that calculates probability distributions using incomplete information [38]. In this study, the MaxEnt (version 3.3.3e) was implemented to predict the probability of *Didymodon* distributions in Tibet.

The recommended default values were used for the convergence threshold (10^−5^), with the maximum number of iterations (500) and 10,000 background points. Suitable regularization values were automatically selected by the program. The selection of environmental variables or functions was carried out automatically under the default rules, which depend on the number of presence records. The default logistic output of MaxEnt is a set of continuous probability values ranging from 0 to 1, where high values indicate a higher relative suitability for a species distribution.

Executing the MaxEnt model involved two procedures: (1) the model was run on the full set of *Didymodon* occurrence data, taking advantage of all available data in order to provide the best estimation of the potential species distribution and the relative importance of the environmental variables; and (2) 10 random partitions of the occurrence data were created by randomly selecting 70% of the occurrence data for training and 30% for testing. The model was run based on each partition.

### Model evaluation and statistical analysis

To evaluate the accuracy of the model predictions, we used both threshold-independent and threshold-dependent methods: (1) AUC (area under curve) is a threshold-independent method that is considered to be an effective indicator of modeling performance independent of the threshold probability [39,40]. The AUC method produces values between 0 and 1, where 1 indicates a perfect fit for the model, 0.9 or higher represents excellent model performance, and 0.5 suggests randomness [41,42]; and (2) TSS (true skill statistic) is a threshold-dependent method in which produced values ranging from -1 to 1, where 1 indicates a perfect fit and value of 0 or less indicates a performance no better than random [43]. The TSS was defined as:

TSS = Sensitivity+Specificity-1 (5)

The AUC and TSS were calculated and created using two output values that were extracted from the 10 random partitions. One value is the observed occurrence value (0 for pseudo-absence points or 1 for test presence points), the other is the predicted value from the logistic output of the MaxEnt model. The final AUC and TSS_max_ (the maximum TSS) values produced are the average values of the 10 replicates evaluated. The evaluation statistics were implemented in the MaxEnt model with an independent training dataset, using the presence-absence package in R 2.14.0 (Available from http://www.R-project.org). The jackknife test was applied to diagnose the relative importance of environmental variables that could potentially contribute to the species distribution model [44]. The environmental variable with the highest training gain when used in isolation is considered to contain the most predictive ability of any variables. Response curves were plotted to demonstrate how variables affect the probability of *Didymodon* presence in the study area, which used all point localities and the respective environmental variable in isolation. Both jackknife test and response curves are available options in MaxEnt.

## Results

### Species diversity

We found 983 *Didymodon* specimens in 181 sample plots. A total of 22 species were identified (Table 3), which comprises approximately 19.47% of the Pottiaceae species in the study area. *Didymodon constrictus* var. *constrictus* was the most dominant species in the study area, with an importance value of 27.909. *D*. *rigidulus* var. *subulatus* had not been previously recorded in China, whereas *D*. *anserinocapitatus* was on the first red list of endangered bryophytes in China.

**Table 3:** *Didymodon* species identified in Tibet, and their relative frequency, coverage, and importance value.

No.	Species	Relative Frequency	Relative Coverage	Importance value
**S1**	*Didymodon constrictus* var. *constrictus*	25.969	29.848	27.909
**S2**	*Didymodon tectorus*	9.948	6.797	8.372
**S3**	*Didymodon constrictus* var. *flexicuspis*	7.539	3.913	5.726
**S4**	*Didymodon perobtusus*	4.817	1.969	3.393
**S5**	*Didymodon rigidulus* var. *rigidulus*	4.712	3.542	4.127
**S6**	*Didymodon tophaceus*	4.712	2.935	3.824
**S7**	*Didymodon rigidulus* var. *icmadophilus*	4.084	2.551	3.318
**S8**	*Didymodon nigrescens*	3.874	2.423	3.149
**S9**	*Didymodon michiganensis*	3.351	1.726	2.539
**S10**	*Didymodon rigidulus* var. *ditrichoides*	2.827	1.375	2.101
**S11**	*Didymodon vinealis*	2.094	1.701	1.898
**S12**	*Didymodon rigidulus* var. *gracilis*	1.780	1.100	1.440
**S13**	*Didymodon asperifolius*	1.152	0.729	0.940
**S14**	*Didymodon rigidulus* var. *subulatus*	1.152	0.492	0.822
**S15**	*Didymodon ferrugineus*	0.942	1.061	1.002
**S16**	*Didymodon fallax*	0.628	0.345	0.487
**S17**	*Didymodon giganteus*	0.524	0.480	0.502
**S18**	*Didymodon rivicolus*	0.524	0.301	0.412
**S19**	*Didymodon rufidulus*	0.209	0.850	0.530
**S20**	*Didymodon anserinocapitatus*	0.105	0.026	0.065
**S21**	*Didymodon japonicus*	0.105	0.032	0.068
**S22**	*Didymodon johansenii*	0.105	0.115	0.110

In order to understand the overall influence of environmental conditions on moss species distribution, we divided the aforementioned 181 sample plots by altitude into six groups, then compared the diversity of species under the different classes of precipitation and altitude (Table 4). Moss species richness differed significantly according elevation and precipitation pattern. Semi-arid areas exhibited the greatest species richness (22 species). Among the different elevation classes, the greatest species diversity was found in the class between 4500 and 5000 m (21 species).

**Table 4:** Number of *Didymodon* species along the altitude gradient and under different precipitation regimes in Tibet.

Altitude (m)	Arid zone	Semi-arid zone	Humid zone	Total
2800–3000	0	4	5	7
3000–3500	0	7	1	7
3500–4000	0	17	2	19
4000–4500	8	13	7	18
4500–5000	16	21	0	21
5000–5600	10	17	0	17
Total	17	22	9	22

### Analysis of environmental heterogeneity

The correlations between species diversity and micro-environmental factors showed that vegetation cover, and altitude were the main environmental factors affecting *Didymodon* diversity in the study area (Table 5). The correlations among micro-environmental factors were generally weak, except for easily interpreted cases such as the correlation between soil moisture at soil depths of 3.8 cm and 7.6 cm (positive), humidity and soil moisture (positive), and the correlation between temperature and altitude (negative), temperature and vegetation type (negative). Humidity and soil moisture at soil depths of 3.8 cm, soil moisture a soil depth of 3.8 cm and soil moisture a soil depth of 7.6 cm were significantly autocorrelated, and thus we included only soil moisture at a soil depth of 3.8 cm below ground in the following analysis of species and habitat micro-environmental factors.

**Table 5:** Correlation of species diversity and environmental factors affecting *Didymodon* in the study area.

	Species diversity	Altitude	Veg-type	Veg-cove	Substrate	Temp	Humidity	Soil-mois
Altitude	0.25							
Veg-type	0.02	0.28**						
Veg-cove	-0.17*	-0.13	0.15					
Substrate	0.14	-0.01	-0.01	-0.19*				
Temp	0.18*	-0.34**	-0.29**	-0.13	-0.07			
Humidity	0.47	-0.01	-0.14	-0.01	-0.01	0.40		
Soil-mois	0.27	-0.10	-0.15	0.02	-0.03	0.48	0.67**	
Soil-mois2	0.31	-0.02	-0.06	0.01	-0.04	0.37	0.72**	0.98**

**Note**:

* and ** represent statistically significant correlations.

*: p < 0.05

**: p < 0.01

Veg-type represents vegetation type, Veg-cove represents vegetation cover, Temp represents temperature, Soil-mois represents soil moisture at a depth of 3.8 cm, and Soil-mois2 represents soil moisture at a depth of 7.6 cm.

**Fig 2:**
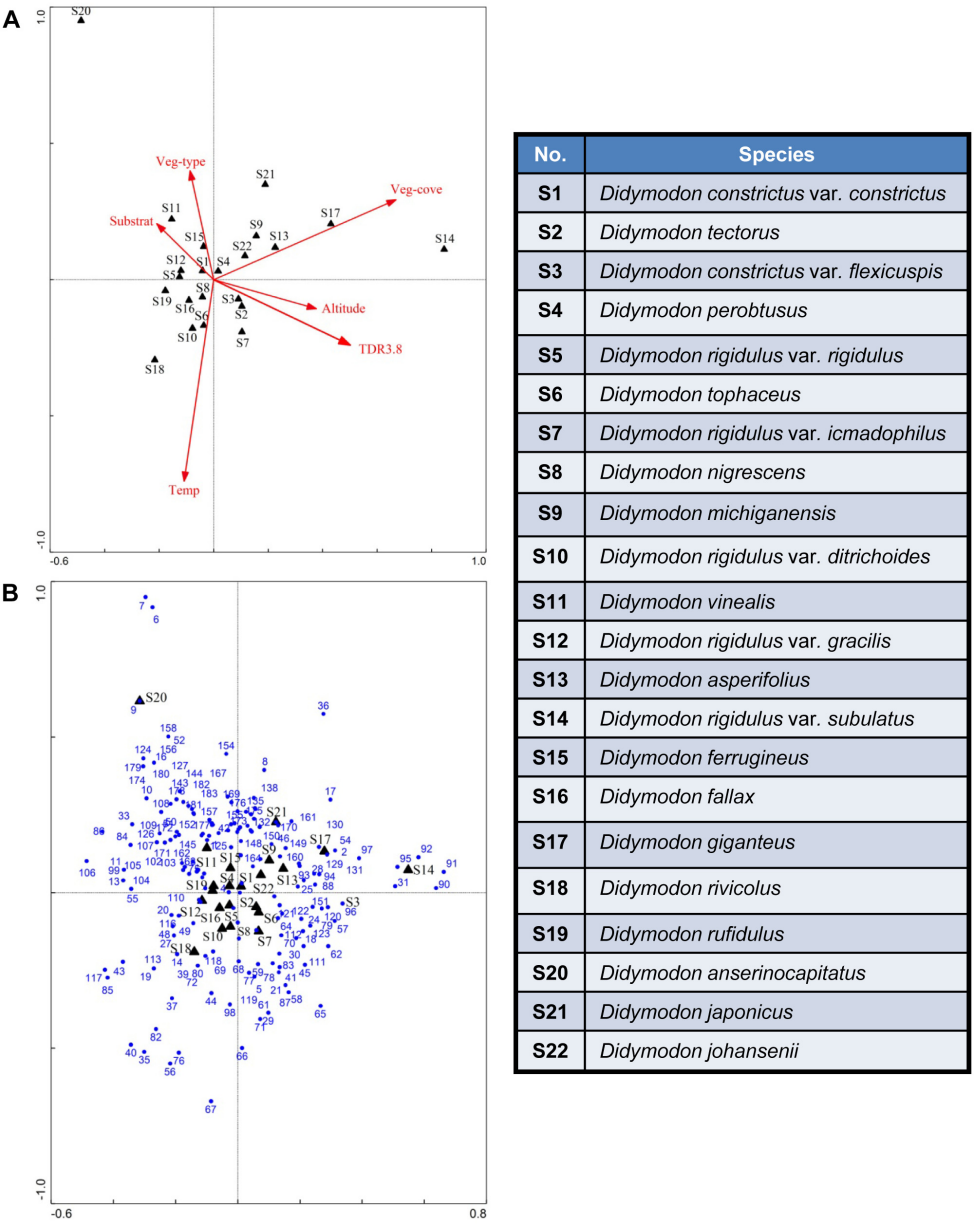
CCA ordination of 22 *Didymodon* species, environmental factors, and sampling plots in the study area. A: CCA ordination of 22 *Didymodon* species and environmental factors; B: CCA ordination of 22 *Didymodon* species and the 181 sampling plots where they were found to grow. The black triangles represent 22 species of *Didymodon*; the blue circles represent the 181 sampling plots where *Didymodon* was found. The red arrows depict environmental factors: Temp represents temperature, Veg-cove represents vegetation cover, Veg-type represents vegetation type, and TDR 3.8 represents soil moisture soil depth of 3.8 cm. S1–S22 refers to *Didymodon* species listed in Table 3.

Analysis of the bryophytes and habitat micro-environmental factors using the 181 plots in CCA showed that altitude, vegetation coverage, and temperature were the key factors influencing the distribution of *Didymodon* (Fig 2). The effects of soil moisture at a depth of 3.8 cm and vegetation type on the distribution of *Didymodon* were minor in comparison to the effects of the other micro-environmental factors. The substrate had the greatest impact on *D*. *constrictus* var. *constrictus* (S1), *Didymodon tectorus* (S2), and *Didymodon constrictus* var. *flexicuspis* (S3) (Fig 2A); further, *D*. *constrictus* var. *constrictus*, *Didymodon perobtusus* (S4), *Didymodon asperifolius* (S13), *Didymodon giganteus* (S17) were widely distributed in plots where vegetation cover was 30–65%. *D*. *anserinocapitatus* (S20) appeared only in shrub vegetation at an elevation of 2748 m. *Didymodon japonicus* (S21), and *Didymodon johansenii* (S22) were found in areas of swamp meadow vegetation at 4787 m and 4800 m altitudes, respectively. *Didymodon rigidulus* var. *subulatus* (S14) was restricted to the alpine meadows of the Nyainqentanglha Mountains and Dagze Mountain at 4800–5200 m altitudes. Other species were primarily distributed in alpine meadows from 3500 to 5600 m (Fig 2B).

### Potential distribution of *Didymodon*

**Fig 3:**
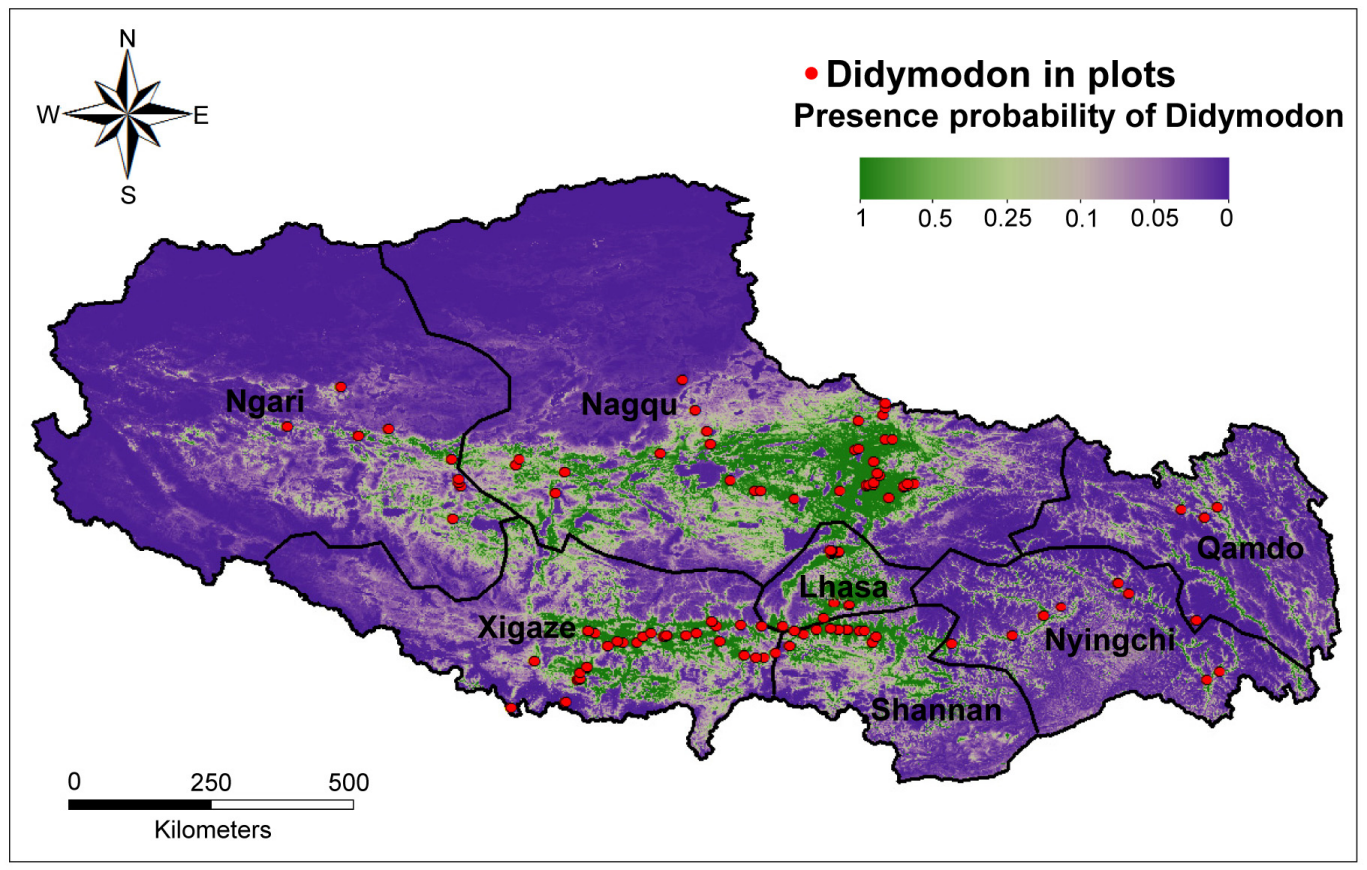
The presence probability of *Didymodon* spatial distributions in Tibet. The red circles represent the *Didymodon* species in the plots that were investigated.

Using the species localities, environmental variables, and the MaxEnt model, we generated maps of the spatial distribution of *Didymodon* in Tibet (Fig 3). The distribution probability was divided into three classes according to the fractional predicted area: most areas are blue, indicating a rare probability (less than 0.05), violet to light green indicates a low to medium probability (0.05–0.25), and dark green indicates a high probability of distribution (0.5–1.0). Fig 3 also clearly shows that the semi-arid regions of Tibet, mainly comprising Nagqu, Xigaze, and Lhasa, had higher distribution probabilities than the other two regions, with Nagqu exhibiting a larger distribution area than the other regions. The AUC value of the threshold-independent method was 0.90, and the TSS_max_ value of the threshold-dependent method was 0.66. These values indicate exceptionally high discrimination of the variation in environmental variables across the confirmed *Didymodon* habitats.

### Environmental variables affecting the distribution of *Didymodon* at macro spatial scales

**Fig 4:**
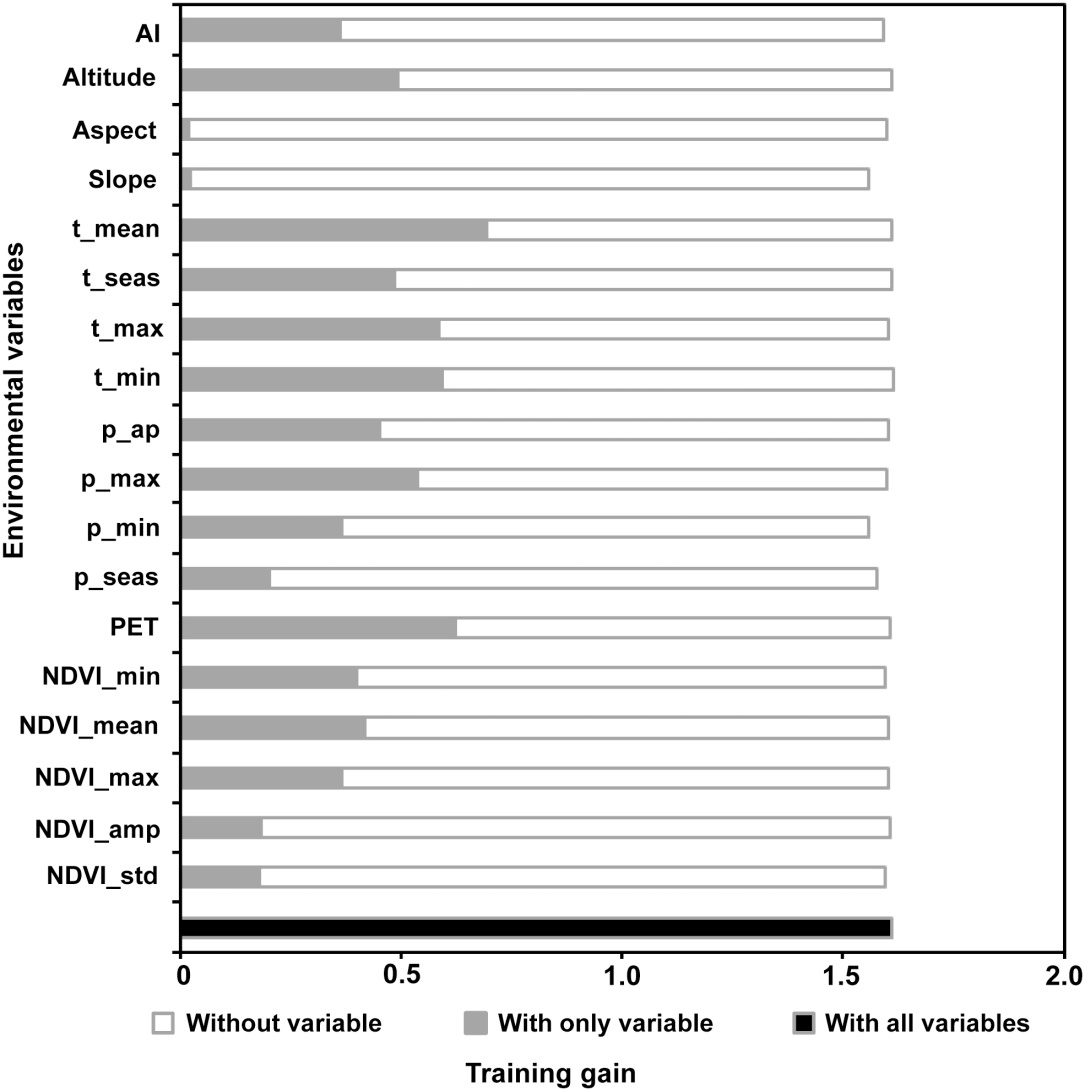
The importance of 22 environmental variables in modeling the distribution of *Didymodon* in Tibet. The training gain describes how much better the MaxEnt distribution fits the presence data compared to a uniform distribution. The names and descriptions of environmental variables are listed in Table 2. The white squares represent the effect of removing a single variable from the full model. The gray squares represent the training gains when using only one environmental variable in MaxEnt. The black square represents the training gains when all variables were run in MaxEnt (1.61).

The contributions of environmental variables to *Didymodon* distributions are shown in Fig 4. The analysis revealed that the climatic variables (the annual mean temperature) exhibited the greatest gains (at 0.70) in the MaxEnt model. The variables of potential evapotranspiration (0.62), minimum temperature of the coldest month (0.60), maximum temperature of the warmest month (0.59), and precipitation of the wettest month (0.54) exhibited the high gains in defining *Didymodon* distribution. The topographic variables of slope and aspect exhibited the lowest gains (both at 0.02), indicating almost no contribution in the model.

**Fig 5:**
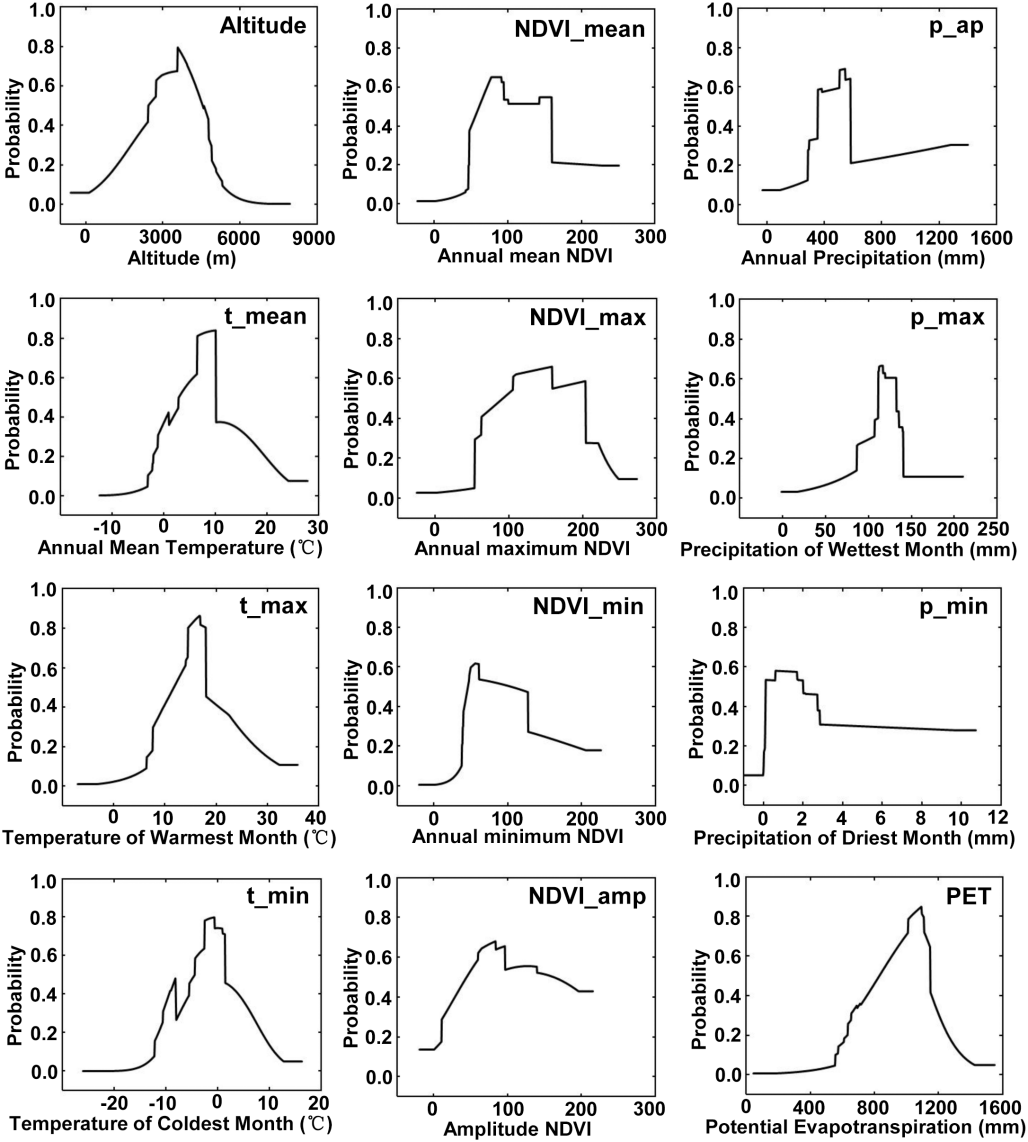
Response curves for the relationship between the probability distributions of *Didymodon* and environmental variables. The curves show the change in the response of *Didymodon* distribution to specific environmental variables.

We examined 12 of the important environmental variables affecting the presence probability of *Didymodon* in the study area (Fig 5). According to the response curves, the presence probability responded positively to altitude, climatic variables, and vegetation variables when a certain value was reached. For example, *Didymodon* exhibited a high probability (over 0.65) of occurrence in plots with altitudes between 3500 and 4200 m, the annual mean temperature was 6.5–10.0°C, and the potential evapotranspiration was 951–1148 mm. When precipitation during the driest month was 0–2 mm, and the annual mean NDVI was 60–160, the possibility of species occurrence was greater than 0.50.

## Discussion

Because of rapid tectonic uplift, Tibet, as the highest plateau in the world, has distinct topography and atmospheric circulation patterns, which in turn greatly affect global climate. The complex climate types found in Tibet create habitat heterogeneity and well-known highland vegetation patterns [45]. In our study, nearly all moss species were collected from the alpine zone. The results showed that species of the genus *Didymodon* are frequently pioneer mosses, able to colonize extreme habitats because of adaptations that enable survival in very harsh environments. This is consistent with the findings of other researchers [15,27].

Studies on moss diversity in northwest Tibet have been almost nonexistent, mainly because of the area’s difficult terrain and traffic restrictions. We accessed these remote areas, particularly northwest Tibet, and conducted our investigation, which involved the exhaustive collection of species data and the discovery of a new record of *Didymodon* species for China. Claudine et al. [9] pointed out that moss diversity changes with altitude and is influenced by microhabitat conditions. Li et al. [13] and Tian et al. [46] also pointed out that the diversity of both specialists and generalists, and the distribution of mosses are positively associated with local habitat and its heterogeneity. Mosses are sensitive to changes in environmental conditions, but are more adaptable to arid desert environments than vascular plants [29].

We illustrated the potential spatial distribution of *Didymodon* in Tibet using the MaxEnt model, which has several advantages over other methods. For example, this model can perform modeling with spatially biased data and limited species occurrence records [47,48]; in addition, it can perform modeling analyses with presence-only points and conduct a built-in jackknife test, which allows for the estimation of the significance of individual environmental variables when computing species distribution [47,49]. The model in this study performed very well, generating high values of AUC and TSS_max_.

Based on species diversity and environmental heterogeneity, we predicted the spatial distribution of *Didymodon* in Tibet. However, there is the potential for deviation when using the MaxEnt model for this purpose, given that our sampling plots were mainly in the semi-arid regions and thus the environmental variables may not be sufficient to accurately describe the habits relevant to species distribution; this may lead to an imprecise prediction of habitat suitability for *Didymodon* [38].

In this study, we measured species diversity, predicted the spatial distribution of *Didymodon*, and analyzed its relationship to environmental heterogeneity in Tibet for the first time. We obtained three primary results. First, a total of 22 species of *Didymodon* was identified in Tibet. Of these, *D*. *rigidulus* var. *subulatus* is a new record for China, while *D*. *constrictus* var. *constrictus* is the dominant species. Second, *Didymodon* had higher distribution probabilities in the semi-arid regions than in the arid and humid regions of Tibet. Third, climatic variables were the main impact factors affecting the distribution of *Didymodon*. These findings are essential for the effective conservation of mosses in Tibet, not only with respect to estimating the species distribution ranges of *Didymodon*, but also for identifying the environmental factors limiting moss distribution, and even monitoring climate change. Our findings also indicate that climate change in Tibet should be given further attention.

## Supporting Information

S1 File Data matrices used to analyze the relationship between *Didymodon* diversity and habitat properties. *Didymodon* matrix contains species names and their coverage in the sample plots (Table A). Environmental data matrix includes all relevant environmental information for quadrats in which *Didymodon* mosses were present (Table B).
(PDF)
