# Supplementary material for: Impacts of Environmental Heterogeneity on Moss Diversity and Distribution of Didymodon (Pottiaceae) in Tibet, China
Source: PLoS One. 2015 Jul 16;10(7):e0132346. doi: 10.1371/journal.pone.0132346 (PMC4504491; doi:10.1371/journal.pone.0132346)
Supplement: S1 File — Didymodon matrix contains species names and their coverage in the sample plots (Table A). Environmental data matrix includes all relevant environmental information for quadrats in which Didymodon mosses were present (Table B). (PDF) [file pone.0132346.s001.pdf]

Supporting Information

S1 File. Data matrices used to analyze the relationship between *Didymodon* diversity and habitat properties. *Didymodon* matrix contains species names and their coverage in the sample plots (**Table A**). Environmental data matrix includes all relevant environmental information for quadrats in which *Didymodon* mosses were present (**Table B**).

Table A. *Didymodon* matrix used in canonical correspondence analysis.

**No.**	**S1**	**S2**	**S3**	**S4**	**S5**	**S6**	**S7**	**S8**	**S9**	**S10**	**S11**	**S12**	**S13**	**S14**	**S15**	**S16**	**S17**	**S18**	**S19**	**S20**	**S21**	**S22**
**1**	0	0	0	0	0	0	0	0	0	0	0	0	1	0	0	0	2	0	0	0	0	0
**2**	2	0	0	0	0	0	0	0	0	0	0	0	0	0	0	0	0	0	0	0	0	0
**3**	5	0	15	0	0	0	0	0	0	0	0	0	40	0	0	0	0	0	0	0	0	0
**4**	34	0	0	0	0	0	0	0	0	0	0	0	0	0	0	0	0	0	0	0	0	0
**5**	22	0	1	0	0	0	0	0	2	0	0	0	0	0	30	0	4	5	0	0	0	0
**7**	0	0	0	0	0	0	0	0	0	0	6	0	0	0	0	0	0	0	0	0	0	0
**8**	2	0	0	0	0	0	0	0	0	0	0	0	0	0	0	0	0	0	0	0	0	0
**10**	0	0	0	0	0	0	0	0	0	0	0	0	5	0	0	0	15	0	0	0	0	0
**11**	13	0	0	0	0	0	0	0	0	0	0	0	0	0	0	0	0	0	0	4	0	0
**12**	0	0	3	0	0	0	0	0	0	0	0	0	0	0	0	0	0	0	0	0	0	0
**13**	28	0	10	0	0	0	0	0	0	0	4	0	0	0	0	0	0	0	0	0	0	0
**14**	1	0	0	0	0	0	0	0	0	0	0	0	0	0	0	0	0	0	0	0	0	0
**15**	0	0	0	0	20	0	0	0	0	0	0	0	0	0	0	0	0	0	0	0	0	0
**16**	10	0	0	0	0	30	0	0	0	0	0	0	0	0	0	0	0	0	0	0	0	0
**17**	26	0	25	0	0	8	0	0	0	0	0	0	0	0	0	0	0	0	0	0	0	0
**18**	15	0	2	1	0	0	0	0	0	0	0	0	0	0	60	0	0	0	0	0	0	0
**19**	0	0	15	0	0	0	0	0	0	0	0	0	0	0	0	0	0	0	0	0	0	0
**20**	21	25	0	0	0	0	0	11	20	0	0	0	0	0	0	0	0	0	0	0	0	0

**21**	30	0	0	0	0	16	15	0	0	0	0	0	0	0	3	0	0	18	0	0	0	0
**22**	2	35	0	0	15	0	0	25	0	0	0	0	0	0	0	0	0	0	0	0	0	0
**23**	45	0	0	0	0	0	0	4	0	0	0	0	0	0	0	0	0	0	0	0	0	0
**24**	0	0	0	0	0	12	0	8	0	0	0	0	0	0	0	0	0	0	0	0	0	0
**25**	25	0	0	3	0	0	12	18	0	0	0	0	0	0	0	0	0	3	0	0	0	0
**26**	0	0	0	2	1	0	0	0	0	0	0	0	0	0	0	0	0	0	0	0	0	0
**27**	26	12	0	15	0	0	6	10	0	0	0	0	7	0	0	0	0	0	0	0	0	0
**28**	5	0	0	0	0	8	0	0	0	0	0	0	0	0	0	0	0	0	0	0	0	0
**29**	0	4	0	0	1	2	20	0	1	0	0	0	0	0	1	0	0	0	0	0	0	0
**30**	21	5	0	1	0	0	0	0	0	0	0	0	0	0	0	0	0	0	0	0	0	0
**31**	7	0	0	1	0	17	0	1	7	0	0	0	0	0	0	10	0	0	0	0	0	0
**32**	0	0	2	0	0	3	0	0	0	0	23	0	0	0	0	0	0	0	2	0	0	0
**33**	6	0	2	0	0	0	0	0	0	0	0	0	0	0	0	0	0	0	0	0	0	0
**34**	0	0	0	0	2	0	0	0	0	0	0	0	0	0	0	0	0	0	0	0	0	0
**35**	1	0	0	0	0	0	0	0	0	0	0	0	0	0	1	0	0	0	0	0	0	0
**36**	0	20	0	1	12	0	2	0	0	0	5	0	0	0	0	0	0	0	12	0	0	0
**37**	0	0	0	0	0	0	0	0	0	0	0	0	1	0	0	0	0	0	0	0	0	0
**38**	10	0	1	0	0	0	0	0	30	0	0	0	0	0	0	0	0	0	2	0	0	0
**39**	30	0	0	0	0	0	0	6	0	0	0	0	0	0	0	0	0	0	0	0	0	0
**40**	20	0	0	0	40	0	0	13	0	0	15	0	0	0	0	0	5	0	0	0	0	0
**41**	30	0	0	0	0	15	0	0	0	0	0	0	0	0	0	0	0	0	42	0	0	0
**42**	0	3	0	0	0	0	0	0	0	0	0	0	0	0	0	0	0	0	0	0	0	0
**43**	0	35	0	0	0	5	0	10	0	0	0	0	0	0	0	0	0	0	0	0	0	0
**44**	40	0	0	0	0	0	0	0	0	0	0	0	0	0	0	0	0	0	0	0	0	0
**45**	45	0	0	0	0	0	3	0	0	0	10	0	0	0	0	0	0	0	7	0	0	0
**47**	8	18	0	0	15	0	15	3	0	0	0	0	0	0	0	0	0	0	0	0	0	0
**48**	5	0	20	0	0	0	46	0	0	0	0	0	0	0	0	0	0	0	0	0	0	0
**49**	0	0	5	0	0	0	0	0	0	0	2	0	0	0	0	0	0	0	0	0	0	0

**50**	15	0	12	0	25	0	15	0	20	0	0	0	0	0	0	0	0	0	0	0	0	0
**51**	15	0	4	0	10	40	0	0	0	0	0	0	0	0	0	0	0	0	0	0	0	0
**52**	30	0	4	0	25	5	0	0	0	0	0	0	0	0	0	0	0	0	0	0	0	0
**53**	0	0	0	0	65	0	0	0	0	0	0	0	0	0	0	0	0	0	0	0	0	0
**54**	1	0	2	0	1	0	0	0	0	0	0	0	0	0	0	0	0	0	0	0	0	0
**55**	0	0	0	0	0	0	0	0	0	0	10	0	0	0	0	0	0	0	0	0	0	0
**56**	15	30	0	0	6	0	0	25	0	0	0	0	0	0	0	0	0	0	0	0	0	0
**57**	9	3	5	0	1	0	0	0	1	2	1	0	0	0	0	0	1	0	0	0	0	0
**59**	25	10	0	5	5	2	0	2	5	0	0	0	0	0	0	0	0	0	0	0	0	0
**60**	7	2	0	0	1	1	0	7	7	0	0	0	0	0	1	0	0	0	0	0	0	0
**61**	0	3	0	0	0	0	0	0	0	0	0	0	0	0	0	0	0	0	0	0	0	0
**62**	16	0	9	0	0	0	0	0	1	0	0	0	0	0	0	0	0	0	0	0	0	0
**63**	24	3	14	0	4	0	1	0	1	0	1	0	3	0	0	0	0	0	1	0	0	0
**64**	0	2	0	0	0	0	0	1	4	2	0	0	0	0	0	0	0	0	0	0	0	0
**65**	1	1	30	0	5	9	3	1	1	2	0	0	2	0	0	0	0	0	0	0	0	0
**66**	8	0	9	0	0	0	0	0	0	1	0	0	28	0	35	0	0	0	0	0	0	0
**67**	20	0	2	0	0	0	0	0	0	3	14	0	0	0	0	0	0	0	0	0	0	0
**68**	21	3	5	0	0	1	12	13	0	5	0	0	0	0	0	0	0	1	0	0	0	0
**69**	0	33	0	0	0	20	0	14	0	0	0	0	0	0	0	0	0	0	0	0	0	0
**70**	1	1	0	0	0	0	0	0	0	0	0	0	0	0	0	0	0	0	0	0	0	0
**72**	0	0	0	3	0	0	0	0	0	0	0	0	0	0	0	0	0	0	0	0	0	0
**73**	29	2	16	3	8	1	0	7	0	2	0	1	1	0	0	0	0	0	0	0	0	0
**75**	15	11	1	0	2	1	0	0	0	0	15	0	0	0	0	0	0	0	0	0	0	0
**76**	5	11	0	20	0	1	0	0	0	0	0	0	0	0	0	0	0	0	0	0	0	0
**77**	43	6	6	0	0	0	0	0	3	6	0	0	0	0	0	0	0	0	0	0	0	0
**78**	45	0	0	0	0	0	0	0	0	0	0	0	0	0	0	0	0	0	0	0	0	0
**79**	2	0	0	0	0	0	0	0	0	0	0	0	0	0	0	0	0	0	0	0	0	0
**81**	1	0	0	0	0	0	0	0	0	0	0	0	0	0	0	0	0	0	0	0	0	0

**84**	40	1	0	0	0	1	0	8	0	15	5	0	1	0	0	0	0	0	0	0	0	0
**85**	2	6	0	0	1	1	0	0	0	10	0	0	0	0	0	0	0	0	0	0	0	0
**88**	2	0	0	0	0	0	0	0	0	0	0	0	0	0	0	0	0	0	0	0	0	0
**89**	31	3	7	0	0	1	1	0	0	0	0	0	0	0	0	0	0	0	0	0	0	0
**90**	26	12	10	3	4	3	9	0	3	1	0	3	0	0	2	1	0	0	0	0	0	0
**92**	2	0	0	0	0	0	0	0	0	1	0	0	0	0	0	0	0	0	0	0	0	0
**94**	33	14	11	6	2	0	13	5	0	5	0	8	3	0	0	0	0	3	0	0	0	0
**95**	4	0	14	0	2	0	0	0	0	0	0	5	0	0	0	0	0	0	0	0	0	0
**96**	12	29	5	0	1	0	13	0	0	0	0	0	0	0	0	0	0	0	0	0	0	0
**97**	0	0	0	0	0	0	0	0	0	0	0	2	0	0	0	0	0	0	0	0	0	0
**98**	32	7	18	0	0	0	0	0	0	9	0	26	0	0	0	0	0	0	0	0	0	0
**101**	1	0	0	0	0	0	0	0	0	0	0	0	0	0	0	0	0	0	0	0	0	0
**103**	10	39	0	0	0	0	0	0	0	0	0	0	0	0	0	0	0	0	0	0	0	0
**104**	0	9	4	0	0	0	0	3	0	0	0	0	15	0	0	0	0	0	0	0	0	0
**105**	5	16	0	0	0	0	3	0	2	2	0	1	3	0	0	0	0	0	0	0	0	0
**106**	12	3	5	0	0	0	1	0	0	0	0	0	0	9	0	0	15	0	0	0	0	0
**107**	5	0	8	1	0	0	0	0	0	0	0	0	0	17	3	0	0	0	0	0	0	0
**108**	7	8	0	0	0	0	0	0	0	0	0	0	0	22	0	0	0	0	0	0	0	0
**109**	17	8	8	0	0	0	1	0	0	0	0	0	0	4	0	0	0	0	0	0	0	0
**110**	13	13	0	0	0	0	10	0	13	0	0	4	0	8	0	0	0	0	0	0	0	0
**111**	8	4	0	0	0	0	0	0	0	0	0	0	0	0	0	0	0	0	0	0	0	0
**112**	4	4	0	0	0	0	12	0	0	17	0	0	0	0	0	0	0	0	1	0	0	0
**113**	44	24	0	3	0	0	0	0	2	0	0	0	0	0	0	0	0	0	0	0	0	0
**114**	0	3	0	0	0	21	0	0	0	0	0	0	0	0	0	0	0	0	0	0	0	0
**115**	49	1	2	0	2	3	3	0	0	2	0	5	0	0	0	0	0	0	0	0	0	0
**116**	21	0	0	0	0	2	0	8	0	0	1	7	0	0	0	0	0	0	8	0	0	0
**117**	77	16	0	0	0	0	0	0	0	0	0	0	0	0	0	0	0	0	0	0	0	0
**118**	86	0	0	0	0	0	0	0	0	0	0	0	0	0	0	0	0	0	0	0	0	0

**119**	0	10	0	0	0	0	0	0	0	0	0	0	0	0	0	0	0	0	0	0	0	0
**120**	0	0	0	0	0	0	0	0	0	16	0	0	0	0	0	0	0	0	0	0	0	0
**121**	4	0	0	0	0	0	0	0	0	0	0	0	0	0	0	0	0	0	0	0	0	0
**122**	0	13	0	0	0	0	0	0	0	0	0	0	0	0	0	0	0	0	0	0	0	0
**123**	0	5	0	0	0	0	0	0	0	0	0	0	0	0	0	0	0	0	8	0	0	0
**125**	0	21	0	0	0	0	0	5	0	0	0	0	0	0	0	5	0	0	0	0	0	0
**126**	0	0	0	0	8	0	0	0	0	0	0	0	0	0	0	0	0	0	0	0	0	0
**127**	0	0	0	0	0	0	0	0	0	0	0	0	0	0	0	28	0	0	0	0	0	0
**128**	0	0	0	0	0	0	0	0	0	0	0	0	0	0	0	5	0	0	0	0	0	0
**129**	8	0	0	0	0	0	0	0	0	0	0	0	0	0	0	0	0	0	0	0	0	20
**130**	0	0	0	9	0	0	0	0	0	0	0	0	0	0	0	0	0	0	0	0	0	0
**131**	0	0	0	33	0	0	0	0	0	0	0	0	0	0	0	0	0	0	0	0	0	0
**132**	78	0	0	0	0	0	0	0	0	0	0	0	0	0	0	0	0	0	0	0	0	0
**133**	76	0	0	7	0	0	0	0	0	0	0	0	0	0	0	0	0	0	0	0	0	0
**134**	91	0	0	0	0	0	0	0	0	0	0	0	0	0	0	0	0	0	0	0	0	0
**135**	1	0	0	26	0	0	0	0	0	31	0	0	0	0	0	0	0	0	0	0	0	0
**136**	0	0	0	5	0	8	0	0	0	0	0	0	0	0	0	0	0	0	0	0	0	0
**137**	0	0	0	0	0	0	0	3	0	0	0	13	0	0	0	0	0	0	0	0	0	0
**138**	24	8	0	0	0	0	0	4	0	0	0	0	0	0	0	0	0	0	4	0	0	0
**139**	44	0	0	0	0	0	0	0	0	0	0	0	0	0	0	0	0	0	0	0	0	0
**140**	36	0	0	0	0	0	0	0	0	0	0	0	0	0	0	0	0	0	0	0	0	0
**141**	24	8	0	0	0	0	0	4	0	0	0	0	0	0	0	0	0	0	4	0	0	0
**142**	28	0	0	0	0	0	0	0	0	0	0	0	0	0	0	0	0	0	0	0	0	0
**145**	44	0	0	0	0	0	0	0	0	0	0	12	0	0	0	0	0	0	0	0	0	0
**146**	0	0	0	0	0	0	0	4	0	0	0	0	0	0	0	0	0	0	0	0	0	0
**147**	20	0	0	0	0	0	0	0	0	0	0	0	0	0	0	0	0	0	0	0	0	0
**149**	0	36	0	0	0	0	0	0	0	0	0	0	0	0	0	0	0	0	0	0	0	0
**150**	0	0	0	0	0	28	0	0	0	0	0	0	0	0	0	0	0	0	0	0	0	0

**162**	0	0	0	0	25	0	0	0	0	0	0	0	0	0	0	0	0	0	0	0	0	0
**163**	60	0	0	0	0	0	0	0	0	0	0	0	0	0	0	0	0	0	0	0	0	0
**164**	17	0	0	21	0	0	0	0	0	0	0	0	0	0	5	0	0	0	0	0	0	0
**165**	0	0	11	0	0	0	0	0	0	0	0	0	0	0	0	0	0	0	0	0	0	0
**166**	15	0	25	0	0	0	0	0	0	0	0	0	0	0	0	0	0	0	0	0	0	0
**167**	74	0	0	0	0	0	0	0	0	0	0	0	0	0	0	0	0	0	0	0	0	0
**168**	28	0	0	0	0	0	0	0	57	0	0	0	0	0	0	0	0	0	0	0	5	0
**169**	33	0	0	0	0	0	0	10	10	0	0	0	0	0	0	0	0	0	0	0	0	0
**170**	0	0	0	0	0	0	0	0	0	0	0	33	0	0	0	0	0	0	0	0	0	18
**171**	61	0	0	0	0	0	0	0	0	0	0	0	0	0	0	0	0	0	0	0	0	0
**172**	13	0	0	13	0	0	0	0	0	0	0	0	0	0	0	0	0	0	0	0	0	0
**173**	13	0	0	0	0	0	0	0	0	0	0	0	0	0	0	0	0	0	0	0	0	0
**174**	23	1	0	0	0	0	0	7	0	0	0	0	0	0	0	0	0	0	0	0	0	0
**175**	18	0	0	0	0	0	0	0	0	0	0	0	0	0	0	0	0	0	0	0	0	0
**177**	33	0	0	31	0	0	0	0	0	0	0	0	0	0	0	0	0	0	0	0	0	0
**178**	0	10	0	0	0	0	0	0	0	0	0	0	0	0	0	0	0	0	0	0	0	0
**179**	20	8	0	7	0	10	0	0	0	0	0	0	0	0	0	0	0	0	0	0	0	0
**181**	33	0	0	0	0	0	0	0	0	0	0	0	0	0	0	0	0	0	0	0	0	0
**182**	50	0	0	13	0	13	0	0	0	0	0	0	0	0	0	0	0	0	0	0	0	0
**183**	0	0	0	0	0	10	0	0	0	0	0	0	0	0	0	0	0	0	0	0	0	0
**184**	13	0	0	0	0	0	0	0	0	0	0	0	0	0	0	0	0	0	0	0	0	0
**185**	0	0	0	0	0	5	0	0	0	0	0	0	0	0	0	0	0	0	0	0	0	0
**186**	0	0	0	0	0	0	0	0	3	0	0	0	0	0	0	0	0	0	0	0	0	0
**187**	0	0	0	3	0	0	0	0	0	0	0	0	0	0	0	0	0	0	0	0	0	0
**188**	0	0	0	5	0	0	0	0	0	0	0	0	0	0	0	0	0	0	0	0	0	0
**189**	0	0	0	0	0	0	0	0	13	0	0	0	0	0	0	0	0	0	0	0	0	0
**190**	13	0	0	0	0	0	0	0	0	0	0	0	0	0	0	0	0	0	0	0	0	0
**191**	0	0	0	0	0	0	0	0	0	0	33	0	0	0	0	0	0	0	0	0	0	0

**192**	13	0	0	0	0	0	0	0	0	0	0	0	0	0	0	0	0	0	0	0	0	0
**193**	18	0	0	0	0	0	0	0	0	0	0	0	0	0	0	0	0	0	0	0	0	0
**194**	0	0	0	0	0	0	0	0	0	0	0	8	0	0	0	0	0	0	0	0	0	0
**195**	0	0	0	0	0	0	0	0	3	0	0	0	0	0	0	0	0	0	0	0	0	0
**196**	13	0	0	0	0	0	0	0	0	0	0	0	0	0	0	0	0	0	0	0	0	0
**197**	18	0	0	0	0	0	0	0	0	0	0	0	0	0	0	0	0	0	0	0	0	0
**198**	0	4	0	0	0	0	0	0	0	0	0	0	0	0	0	0	0	0	0	0	0	0
**199**	8	0	0	0	0	0	0	0	0	0	0	0	0	0	0	0	0	0	0	0	0	0
**200**	4	0	0	0	0	0	0	0	0	0	0	0	0	0	0	0	0	0	0	0	0	0
**201**	16	0	0	0	0	0	0	0	0	0	0	0	0	0	0	0	0	0	0	0	0	0
**202**	20	0	0	0	0	0	0	0	0	0	0	0	0	0	0	0	0	0	0	0	0	0
**203**	4	0	0	0	0	0	0	0	0	0	0	0	0	0	0	0	0	0	0	0	0	0
**204**	8	0	0	0	0	0	0	0	0	0	0	0	0	0	0	0	0	0	0	0	0	0
**206**	0	0	0	4	0	0	0	0	0	0	0	0	0	0	0	0	0	0	0	0	0	0
**207**	0	0	0	0	0	12	0	0	0	0	0	0	0	0	0	0	0	0	0	0	0	0
**209**	0	0	0	0	0	0	0	0	0	0	0	0	16	0	0	0	0	0	0	0	0	0
**211**	8	0	0	0	0	0	0	0	0	0	0	0	0	0	0	0	0	0	0	0	0	0
**212**	0	0	0	0	0	0	0	0	0	0	4	0	0	0	0	0	0	0	0	0	0	0
**214**	36	0	0	0	0	0	0	0	0	0	0	0	0	0	0	0	0	0	0	0	0	0
**215**	0	0	0	0	0	0	0	0	0	0	36	0	0	0	0	0	0	0	0	0	0	0
**216**	0	1	0	0	0	0	0	0	0	0	0	0	0	0	0	0	0	0	0	0	0	0
**217**	0	0	0	0	0	0	0	0	0	0	0	0	0	0	0	5	0	0	0	0	0	0
**218**	20	0	0	0	0	0	0	0	0	0	0	0	0	0	0	0	0	0	0	0	0	0
**220**	0	0	0	0	5	0	0	0	0	0	0	0	0	0	0	0	0	0	0	0	0	0
**221**	44	0	0	0	0	0	0	0	0	0	0	0	0	0	0	0	0	0	0	0	0	0

Note: S1–S22 refers to *Didymodon* species listed in Table 3.

Table B. Environmental data matrix used in canonical correspondence analysis.

No.	Altitude	Veg-types	Veg-cover	Substrate	Temp	TDR3.8
1	3994	1	75	1	0	0
2	3764	1	50	1	18	50
3	4362	2	85	1	0	0
4	3254	3	85	1	30	9
5	4090	1	75	1	31	7
7	1849	5	90	1	0	0
8	1725	5	90	1	0	0
10	2725	2	95	1	18	20
11	2748	3	55	1	0	0
12	3880	6	30	1	0	0
13	2934	3	30	1	27	15
14	3017	3	65	1	22	25
15	3163	7	30	1	27	14
16	3729	3	30	1	31	19
17	3573	8	55	1	22	14
18	3582	8	45	1	0	0
19	3550	9	60	1	16	55
20	3674	1	80	1	35	29
21	3755	1	30	1	31	9
22	3799	6	20	1	24	23
23	3821	1	85	1	36	15
24	3932	1	20	1	13	24
25	4140	18	40	2	23	28
26	4022	1	55	1	24	35
27	4589	18	80	2	18	25

28	4418	13	60	1	18	18
29	5185	18	20	2	12	8
30	4419	14	65	1	14	29
31	4613	14	65	1	36	21
32	4818	6	45	1	16	27
33	4996	14	75	1	12	43
34	4289	3	30	1	9	20
35	4321	17	20	0	0	0
36	4379	17	10	1	12	48
37	4955	13	15	0	29	0
38	2890	16	95	1	15	48
39	4037	8	35	1	36	14
40	3895	9	45	1	26	35
41	4089	9	55	1	27	6
42	4382	1	15	1	33	0
43	4461	1	65	1	24	13
44	3665	9	65	1	18	17
45	4078	17	40	1	31	0
47	4370	14	65	1	34	6
48	4277	1	65	1	28	26
49	4175	14	65	1	28	23
50	4074	14	60	1	27	18
51	3980	14	35	1	27	18
52	3879	14	60	1	29	11
53	3776	14	60	1	14	4
54	3738	14	60	1	14	4
55	3697	20	95	1	18	0
56	3697	9	65	1	18	0

57	4575	14	80	1	10	27
59	4570	14	30	1	14	23
60	4628	1	10	1	34	12
61	4612	17	55	1	24	49
62	4641	1	50	1	26	23
63	4745	1	15	2	20	35
64	4634	1	15	2	18	19
65	4725	14	45	1	20	24
66	5227	14	45	1	16	37
67	4581	1	35	1	12	13
68	4679	14	65	1	18	18
69	4573	1	75	1	31	20
70	4585	1	50	1	35	11
72	4605	1	40	1	43	9
73	4699	1	45	1	17	6
75	4690	14	45	1	21	10
76	4773	1	40	1	13	21
77	5078	1	35	1	22	14
78	5273	0	0	0	0	0
79	4870	1	25	1	10	14
81	4959	1	20	1	0	0
84	4644	14	30	2	10	17
85	4628	14	40	1	39	8
88	4506	1	40	1	23	18
89	4930	1	35	1	15	19
90	4556	1	50	1	13	29
92	4639	1	35	1	17	0
94	4590	1	50	1	8	22

95	4585	13	20	1	34	12
96	4473	14	85	1	28	12
97	4454	6	10	2	0	0
98	4445	13	10	2	27	6
101	4450	13	5	2	0	0
103	4321	1	70	1	30	16
104	5325	18	75	1	9	17
105	5283	18	40	2	-4	11
106	5173	14	95	1	11	40
107	5082	14	95	1	10	44
108	4976	14	95	1	8	37
109	4888	3	85	1	4	0
110	4789	3	90	1	4	0
111	4691	1	90	1	7	27
112	4576	14	85	1	20	29
113	4485	14	95	1	14	28
114	4389	1	35	1	30	19
115	5528	18	30	2	-10	0
116	5451	18	30	2	-10	0
117	3695	9	65	1	21	27
118	3602	9	65	1	21	3
119	3569	6	65	1	21	3
120	3583	6	35	1	21	3
121	3581	1	65	3	21	3
122	3565	19	30	1	21	3
123	3565	19	70	1	21	3
125	3739	18	45	2	14	15
126	3783	12	35	2	14	15

127	3783	12	35	2	28	32
128	3922	1	65	1	28	32
129	3921	9	65	1	28	32
130	3919	6	35	2	23	30
131	3919	6	45	2	23	30
132	3849	19	65	1	25	33
133	3853	19	65	1	30	8
134	3853	6	10	1	30	8
135	3853	9	70	1	30	8
136	3853	9	90	1	30	8
137	3853	9	85	1	30	34
138	3851	9	70	3	30	34
139	3896	1	55	3	27	37
140	3943	1	60	1	27	37
141	4013	18	45	2	0	0
142	4546	14	75	1	0	0
145	4342	1	20	2	0	0
146	4543	14	55	1	0	0
147	5055	14	45	1	0	0
149	5031	4	80	1	0	0
150	4798	1	75	1	0	0
162	5058	14	95	1	0	0
163	4987	14	80	1	0	0
164	4595	17	75	1	0	0
165	4581	17	80	1	0	0
166	4591	17	80	1	0	0
167	4820	17	85	1	0	0
168	4779	17	85	1	0	0

169	4659	14	60	1	0	0
170	4787	17	65	1	0	0
171	4719	1	30	5	0	0
172	4732	14	55	1	0	0
173	4663	14	55	1	0	0
174	4616	14	55	5	0	0
175	4832	14	55	5	0	0
177	5031	15	35	1	0	0
178	5182	14	60	1	0	0
179	5253	6	35	1	0	0
181	4658	14	70	1	0	0
182	5061	14	75	1	0	0
183	4911	14	75	1	0	0
184	5570	10	45	2	0	0
185	4525	10	45	1	0	0
186	4268	1	60	1	0	0
187	3697	9	80	1	0	0
188	4600	14	65	1	0	0
189	4560	14	55	1	0	0
190	4500	14	55	1	0	0
191	3697	20	65	1	0	0
192	4950	14	45	1	0	0
193	5200	14	70	1	0	0
194	4700	14	75	1	0	0
195	4900	14	35	1	0	0
196	5000	14	35	1	0	0
197	5100	14	60	1	0	0
198	5150	14	45	1	0	0

199	5250	14	50	1	0	0
200	4300	14	70	1	0	0
201	4730	14	70	1	0	0
202	4370	14	70	1	0	0
203	4720	14	70	1	0	0
204	4710	14	70	1	0	0
206	4590	14	35	1	0	0
207	4670	14	65	1	0	0
209	4480	14	45	3	0	0
211	4600	14	75	1	0	0
212	4480	14	70	1	0	0
214	4668	14	50	1	0	0
215	4570	14	50	4	0	0
216	4300	14	50	6	0	0
217	4400	14	50	1	0	0
218	4390	14	50	1	0	0
220	4300	14	50	1	0	0
221	4370	14	50	2	0	0

Note: Veg-types represents vegetation type, Veg-cover represents vegetation cover, Temp represents temperature, and TDR 3.8 represents soil moisture at a depth of 3.8 cm.
